# Chemosensory Dysfunction in Long-Term COVID-19 Assessed by Self-Reported and Direct Psychophysical Methods

**DOI:** 10.3390/life12101487

**Published:** 2022-09-25

**Authors:** Javier Albayay, Lara Fontana, Valentina Parma, Massimiliano Zampini

**Affiliations:** 1 Center for Mind/Brain Sciences, University of Trento, Corso Bettini 31, 38068 Rovereto, TN, Italy; 2 Monell Chemical Senses Center, 3500 Market Street, Philadelphia, PA 19104, USA

**Keywords:** COVID-19, chemosensory dysfunction, smell, taste, chemesthesis

## Abstract

Chemosensory dysfunction is a frequent postacute sequela of COVID-19. Depending on the type of test used to measure it (self-report vs. direct test), the degree of chemosensory dysfunction in long-term COVID-19 has been found to be highly variable. In this manuscript, we report the cross-sectional data (first assessment) of a longitudinal study (6-month follow-up) examining smell, taste, and chemesthesis in participants affected by long-term COVID-19 (COVID+) and participants without COVID-19 (COVID−) by means of both self-reported and direct psychophysical methods. In total, 208 Italian participants (COVID+ *n* = 133; COVID− *n* = 75) completed the Smell and Taste Check developed by the Global Consortium for Chemosensory Research (GCCR), which includes self-reports on smell, taste, and chemesthetic abilities as well as direct intensity ratings of unstandardized smell, taste, and chemesthetic household items. Furthermore, all participants completed SCENTinel, a validated direct smell test. We found a positive association between the self-reported, unstandardized direct test and the validated direct test for smell, indicating moderate to large agreement across measures. Furthermore, the performance on SCENTinel was significantly associated with self-reported smell loss. A positive association between the self-reports and the intensity of household items was also retrieved for taste and chemesthesis. The time relative to COVID-19 onset (267.3 ± 113.9 days) did not modulate the chemosensory performance of self-reported abilities, intensity ratings, and SCENTinel. All in all, we confirm the impairment of three chemical senses (smell, taste, and chemesthesis) in an independent sample of Italian participants affected by long-term COVID-19 by using and comparing self-reported and direct psychophysical methods. We contribute to the discussion on best practices to monitor chemosensory dysfunction in individuals affected by long-term COVID-19.

## 1. Introduction

Coronavirus disease 19 (COVID-19), caused by SARS-CoV-2 infection, comprises highly variable symptoms across individuals, including fever, shortness of breath, fatigue, body aches, and dry cough [1]. Chemosensory dysfunction, such as sudden smell and taste loss, is recognized as an early hallmark symptom of COVID-19 [2,3,4], which can be present in individuals who are asymptomatic or minimally symptomatic [5]. Across variants up to Delta, ~85% of patients appear to recover smell function within 3 weeks, with ~1 in 7 reporting persistent olfactory dysfunction in the postacute phase of the disease [6]. In a recent study, Coelho et al. [7] surveyed 616,318 individuals in the United States who had contracted COVID-19. The authors found that the odds ratios regarding the possibility of developing chemosensory dysfunction—including smell, taste, and chemesthesis [3,8]—for the Alpha, Delta, and Omicron variants, with the original variant set as the baseline, were 0.5, 0.44, and 0.17, respectively, indicating a reduction in the prevalence of smell loss with Omicron. Nevertheless, given that the highest peak of infections to date during the COVID-19 pandemic was reached during Omicron, smell loss still impacts a large portion of the population [9].

The importance of the chemical senses, namely the olfactory (smell), gustatory (taste), and trigeminal chemosensory systems, is often overlooked due to the dominant role attributed to other sensory modalities, such as vision and audition [10]. Nevertheless, these senses are involved in critical survival functions. For instance, olfaction, the most studied of the chemical senses, plays a major role in appetite regulation, the avoidance of environmental hazards, and social communication [11]. Moreover, chemosensory disorders involve quantitative reduction and qualitative changes in smell or taste [3], which can significantly impact the quality of life, nutrition, and safety [12]. On the one hand, quantitative smell disorders include anosmia and hyposmia (i.e., complete and partial smell loss, respectively), and qualitative disorders include parosmia (i.e., smell distortions) and phantosmia (olfactory hallucinations) [13]. On the other hand, quantitative taste alterations comprise ageusia and hypogeusia (i.e., complete and partial taste loss, respectively), whereas qualitative taste alterations comprise parageusia (i.e., taste distortions) and phantogeusia (i.e., taste hallucinations). The classification of chemesthetic disorders is currently lacking.

The impact of smell and taste loss as clinical consequences of COVID-19 is a current topic of active research [14]. Despite the current consensus on smell and taste alterations as cardinal symptoms of COVID-19, even in the absence of other symptoms [4], few studies have employed direct psychophysical (objective) measures to quantify such symptoms as compared to self-reported (subjective) measures [4,15,16,17,18]. Moreover, it has been shown that self-reported and direct chemosensory measures have a weak correlation between each other [19,20,21], and this has also emerged when considering individuals affected by COVID-19 [4,22,23]. For instance, a review by Trecca et al. [2] indicated that the prevalence of smell alterations ranges from 14 to 89% and the prevalence of taste alterations ranges from 9 to 88% when assessed via self-reports. These prevalences range from 21 to 96% and from 12 to 66%, respectively, when measured by direct psychophysical methods. According to a meta-analysis by Hannum et al. [17], the prevalence of chemosensory dysfunction in individuals affected by COVID-19 reaches 45% (95% CI 31.1–58.5%) when assessed by self-reported methods and reaches 77% (95% CI 61.4–89.2%) when assessed by direct methods.

Both the direct psychophysical and self-reported evaluations possess intrinsic advantages and disadvantages. On the one hand, direct psychophysical tests are the gold standard for assessing olfactory function [24,25]. They allow the quantification of the perception of olfactory stimuli of an individual and can account for the odor detection threshold, odor discrimination, and odor identification screening [26]. Nonetheless, such instruments can be costly and time-consuming to administer. Further, they may require in-person interactions with potentially infectious individuals [24,27]. On the other hand, self-reported measures are typically free, quick to administer, and can be conducted both in person and remotely (e.g., online surveys). However, they cannot guarantee the intended interaction with a chemosensory stimulus [3,6,13]. Nevertheless, the direct evaluation of smell has shown that self-reports can underestimate the prevalence of chemosensory dysfunction in individuals affected by COVID-19 [18]. Furthermore, many individuals are inaccurate in judging both the nature and the degree of such impairments [4], for instance, by reporting taste problems when it is smell that is affected [5]. Thus, self-reported measures might be insufficient by themselves to capture chemosensory dysfunction when not contrasted against direct measures.

The COVID-19 pandemic revealed the need for rapid and low-cost direct smell screening. However, most of the available commercial smell tests do not meet the requirements for population surveillance [27]. Moreover, many of these tests, such as the University of Pennsylvania Smell Identification Test [24] and the Pocket Smell Test [28], focus only on odor identification. It should be noted that whereas Sniffin’ Sticks [25] are often used for the screening of odor identification, the instrument also allows individuals to test odor discrimination and the olfactory threshold. An exclusive screening of odor identification is problematic, not only because it is the olfactory task most sensitive to cognitive deficits but also because it may fail to detect a reduction in the perceived intensity of the odor, which has recently proven to be rather informative [27,29]. Indeed, the rating of the intensity of an odorant, which has not been assessed in commercial smell tests until recently [27], has allowed researchers to identify smell loss during illness as the best predictor of COVID-19, even when assessing chemosensory dysfunction by self-reported measures [6,16,17].

In this study, we aimed to quantify olfactory dysfunction in participants affected by long-term COVID-19 and participants without COVID-19—based on self-reported results of polymerase chain reaction (PCR) tests—by comparing the results of at-home direct psychophysical tests and self-reported methods. We report the cross-sectional data (first assessment) of a longitudinal study (6-month follow-up). We used the Smell and Taste Check developed by the Global Consortium for Chemosensory Research (GCCR), which includes both self-reported measures of subjective experiences and direct intensity ratings postexposure to smell, taste, and chemesthetic stimuli. Furthermore, we used the SCENTinel smell test [27] as a direct measure of odor detection, intensity, and identification (please refer to Section 2.1.1 below). Overall, we expected the direct and self-reported methods to show convergence by means of medium to large correlations across measures. Furthermore, we anticipated a positive association between self-reported chemosensory abilities and experienced intensities (considering both the SCENTinel test and the GCCR Smell and Taste Check). Regarding participants’ COVID-19 status, we expected individuals affected by COVID-19 to report lower chemosensory abilities and reduced experienced intensities compared to participants without COVID-19. Finally, in line with previous research including self-reports [6], we anticipated that the time relative to COVID-19 onset (days since COVID-19 diagnosis) would modulate the chemosensory abilities and experienced intensities in participants affected by COVID-19.

## 2. Materials and Methods

### 2.1. Participants

In total, 208 Italian participants aged between 18 and 74 years old (mean age = 40.9 ± 13.3 years old, 74% women) were recruited by nonprobability convenience sampling considering the following inclusion criteria: (i) adults aged 18 years and older; (ii) tested positive or negative for SARS-CoV-2 by real-time PCR; (iii) access to a smart device (phone or tablet) or a computer; and (iv) fluent Italian speakers. Based on the self-reported results of PCR tests (positive or negative COVID-19 diagnoses), participants were classified as “COVID+” (*n* = 133) or “COVID−” (*n* = 75); the descriptive characteristics of the sample according to COVID-19 status are presented in Table 1. Self-reports on the frequency of smell and taste alterations are reported in Table 2. All participants were informed about the aim and the procedures of the study and gave informed consent. Participants did not receive any monetary compensation. The study was conducted in accordance with the Declaration of Helsinki [30] and was approved by the Ethics Committee of the University of Trento (protocol 2021-015).

#### 2.1.1. SCENTinel Smell Test

Parma et al. [27] developed and validated the SCENTinel rapid smell test (a portmanteau of “scent” and “sentinel”), a low-cost self-administered test for the assessment of three olfactory skills, namely odor detection, odor intensity, and odor identification. SCENTinel is an instrument that meets the criteria for large scale deployment as a smell test for population surveillance (i.e., speed, the use of easily identifiable odorants, the number of odorants, the uniform delivery of odorants, the avoidance of physical contamination, and robustness against guessing). Further, the test has proven to be able to discriminate individuals with smell loss and is likely to be useful in clinical scenarios, including screening for viral infections. The test is comprised of three patches only one of which contains an odorant. To complete the test, participants have to consecutively open one patch at a time, smell each patch, and then reseal it. First, for the odor detection subtests, participants are instructed to indicate which is the patch with the strongest odor. Then, for the odor intensity subtests, participants have to rate the intensity of the odor on a visual analog scale (VAS) ranging from 0 (“no smell”) to 100 (“very strong smell”). Finally, for the odor identification subtests, participants have to select the best verbal and visual labels for the odor among four options. If the participant initially fails to identify the odor, they are instructed to try once again among the three remaining alternatives. We followed the scoring systems described in the original validation of SCENTinel [27] to determine the percentage of participants that failed to meet the accuracy criteria for the overall test as well as the three subtests.

#### 2.1.2. GCCR Smell and Taste Check

We used the Italian version of the Smell and Taste Check developed by the by the GCCR, an online tool that collects both direct and self-reported chemosensory data. On the one hand, the GCCR Smell and Taste Check includes an online survey with questions related to demographics, health, and the quantitative self-reported ability to smell, taste, and experience nasal and oral irritation (chemesthesis). On the other hand, the test includes an active chemosensory task where participants rate the intensity of the smell or taste of four different categories of household items/foods and the intensity of one nasal and one oral irritant. Self-reported abilities and perceived intensities were rated using a 100-point VAS [3,6]. 

### 2.2. Procedures

Participants were recruited through personal contacts of the investigators and dissemination channels such as public message boards and social media. Participants who wanted to join the project contacted the researchers via email (present on the posters advertised on social media). Participants who met the inclusion criteria of this study were then asked for their home address, which was necessary to send the instructions and SCENTinel testing cards. A link was also sent by email to access all the material. The SCENTinel test for the screening of olfactory function was sent by post to their home. The data of the GCCR Smell and Taste Check were collected online via links provided to the participants. The anonymity of the participants was protected, as each participant was provided with an ID known only by the researchers, which was used to complete all the instruments online. Before filling the questionnaires, the participants read the information form, including the description of all the procedures of the study, and the form for the processing of personal data and gave their informed consent online. All the online questionnaire material was presented using the formr framework [31].

### 2.3. Statistical Analyses

We computed separate smell, taste, and chemesthesis composite scores for the perceived smell and taste intensity ratings obtained from the GCCR Smell and Taste Check. The composite scores were the means of the corresponding ratings of all item categories; a minimum of one rating per category was required to include the computed scores for smell and taste. To determine if the COVID+ group exhibited reduced experienced intensity to smell, taste, and chemesthetic stimuli compared to the COVID− group, we carried out analyses of variance (ANOVAs) considering the COVID-19 status (COVID+ group vs. COVID− group) as a between-subjects factor. Moreover, we performed a Chi-squared test to determine whether there were significant differences between both groups regarding the outcome of SCENTinel (total score as well as the odor detection, intensity, and identification subtests). We computed partial eta square (*η*^2^_p_) and Cramér’s V as measures of effect sizes for ANOVAs and Chi-squared tests, respectively. Further, to test if the self-reported chemosensory abilities (smell, taste, and chemesthesis) correlated positively with the experienced intensity of household items, we separately computed Spearman’s rho correlation coefficients (*r*_s_) between the two measures for smell, taste, and chemesthesis. Furthermore, we used Chi-squared test to determine the association between the outcome of SCENTinel and self-reports of smell loss. To determine the association between the extent of chemosensory dysfunction and the time relative to COVID-19 onset (i.e., days since COVID-19 diagnosis), we computed linear regressions and Poisson regressions with the log link function for numeric (VAS ratings) and count data (SCENTinel total score and subtests), respectively. We used a robust (sandwich) variance estimator of the regression parameters [32]. The alpha level was set at 0.05. It should be noted that whereas all participants completed the SCENTinel test, 24 participants in the COVID+ group and 2 participants in the COVID− group did not complete the GCCR Smell and Taste Check and were excluded from the corresponding analyses. We used RStudio (version 1.4.1103, Boston, MA, United States) for all our analyses. For the sake of the openness, transparency, and reproducibility of research, the data reported in this study and the analysis scripts are available in the Open Science Framework repository accessible at https://osf.io/3xbqg/?view_only=5e3b7b32ab9147bc89bb72aefe4b79e5 (accessed on 15 February 2022).

## 3. Results

### 3.1. Chemosensory Abilities and Experienced Intensities

**Figure 1:**
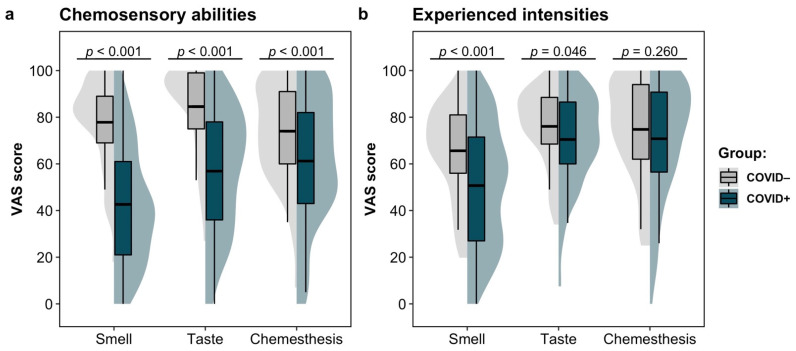
Split violin plots for the GCCR Smell and Taste Check outcomes by COVID-19 status: (**a**) chemosensory abilities and (**b**) the experienced intensity of household items. The horizontal thick line within the boxes represents the mean. The lower and upper horizontal lines indicate the 25th and 75th percentiles of the distribution, respectively (i.e., interquartile range). The whiskers indicate the minimum and maximum values located in a range 1.5 times greater than the interquartile range. The shaded area surrounding each box depicts a rotated kernel density plot.

As shown in Figure 1, the COVID+ group reported lower chemosensory abilities (smell, taste, and chemesthesis) compared to the COVID− group. Moreover, the COVID+ group reported reduced experienced intensities to smell and taste household items compared to the COVID− group. Conversely, the experienced intensity of chemesthetic stimuli did not differ significantly between the two groups. The results of the ANOVAs and the descriptive statistics by group are reported in Table 3.

Fewer participants in the COVID+ group met SCENTinel’s overall accuracy criteria compared to the COVID− group. This pattern was also confirmed in each subtest, namely odor detection, odor intensity, and odor identification (see Table 4). Furthermore, the COVID+ group rated the target odors as less intense (52.6 ± 21.8) compared to the COVID group (73.8 ± 19.2) (*F*(1,188) = 49.5, *p* < 0.001, *η*^2^_p_ = 0.194). It should be noted that the identity of the target odor (*F*(9, 188) = 0.83, *p* = 0.590, *η*^2^_p_ = 0.038) and the interaction odor identity × group (*F*(9, 188) = 1.16, *p* = 0.320, *η*^2^_p_ = 0.053) did not significantly affect the intensity ratings of SCENTinel.

Contrary to our prediction, the regression analyses showed that the time relative to COVID-19 onset (267.3 ± 113.9 days since COVID-19 diagnosis via PCR test) did not modulate the performance of the participants in the COVID+ group on the GCCR Smell and Taste Check or the SCENTinel test. The results of the linear regression (continuous variables) and Poisson regressions (binary variables) are presented in Table 5.

### 3.2. Correspondence between Self-Reported and Direct Psychophysical Chemosensory Measures

**Figure 2:**
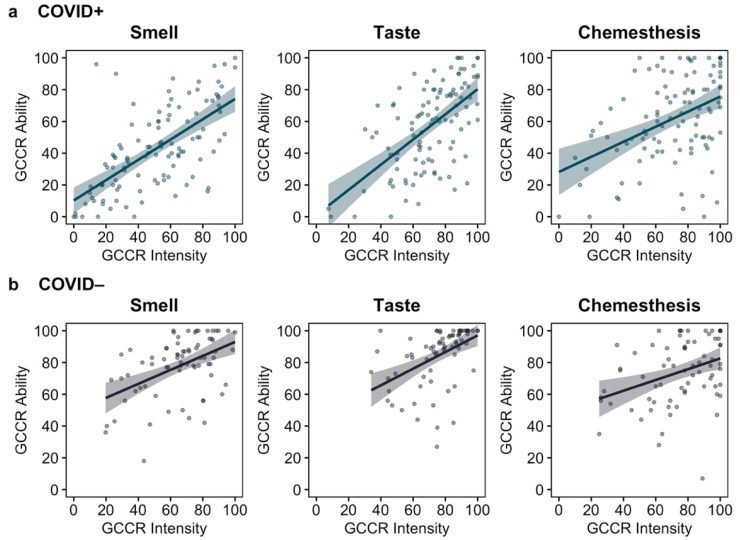
The association (Spearman’s rho) between self-reported smell, taste, and chemesthesis abilities (GCCR ability) and the intensity of unstandardized household smell, taste, and chemesthetic items (GCCR intensity) in (**a**) the COVID+ group (*n* = 109) and (**b**) the COVID− group (*n* = 73).

Overall, we found a significant positive correspondence between the self-reported chemosensory abilities and the intensity of household items (GCCR Smell and Taste Check). In the COVID+ group, the association was strong for smell (*r*_s_ = 0.65, *p* < 0.001), taste *(r*_s_ = 0.58, *p* < 0.001), and chemesthesis (*r*_s_ = 0.41, *p* < 0.001). The association in the COVID− group was also strong for smell *(r*_s_ = 0.43, *p* < 0.001), taste (*r*_s_ = 0.63, *p* < 0.001), and chemesthesis (*r*_s_ = 0.40, *p* < 0.001); see Figure 2.

**Figure 3:**
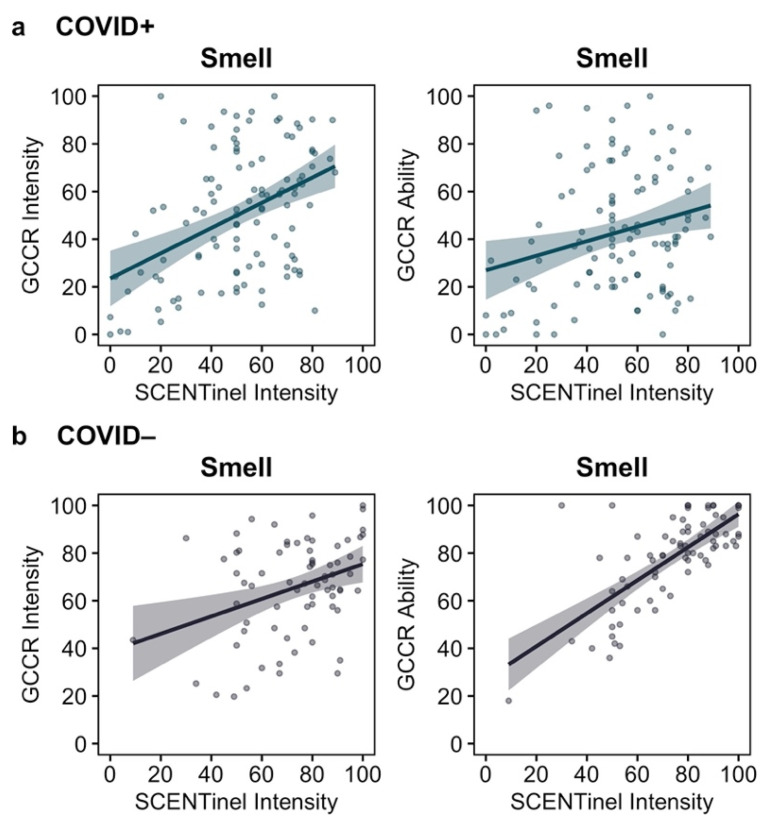
The association (Spearman’s rho) between the intensity of a calibrated odor stimulus (SCENTinel intensity), the intensity of unstandardized household smell items (GCCR intensity), and self-reported smell ability (GCCR ability) in (**a**) the COVID+ group (*n* = 109) and (**b**) the COVID− group (*n* = 73).

Furthermore, we found a moderate positive association between the self-reported, unstandardized direct test (GCCR Smell and Taste Check) and the validated direct test via the odor intensity SCENTinel subtest. The association between the SCENTinel intensity and the GCCR intensity of smell items was moderate for the COVID+ group (*r*_s_ = 0.39, *p* < 0.001) and weak for the COVID− group (*r*_s_ = 0.28, *p* = 0.016). The association between the SCENTinel intensity and the GCCR self-reported smell ability was weak for the COVID+ group (*r*_s_ = 0.21, *p* = 0.030) and strong for the COVID− group (*r*_s_ = 0.64, *p* < 0.001); see Figure 3.

Moreover, the performance on SCENTinel (total score) was significantly associated with self-reported smell loss (78.9% in the COVID+ group), χ^2^(1) = 15, *p* < 0.001, Cramér’s V = 0.267.

## 4. Discussion

In this study, we compared the results of self-reported and direct chemosensory measures in participants affected by long-term COVID-19 and participants without COVID-19. Overall, we confirm in an independent sample the impairment of smell, taste, and chemesthesis in long-term COVID-19. In line with our hypotheses, we found a significant correspondence between self-reported and direct psychophysical measures. Moreover, we found a positive correlation between chemosensory abilities (smell, taste, and chemesthesis) and the experienced intensity of items/foods. When comparing the performance between groups, we found that the COVID+ group showed impaired chemosensory abilities as well as reduced experienced intensities of chemosensory stimuli compared to the COVID− group. Conversely, our hypothesis regarding the predictive values of the time relative to COVID-19 onset for the chemosensory abilities and experienced intensities of participants affected by COVID-19 was not retrieved.

While the importance attributed to chemical senses is highly variable [33,34], the COVID-19 pandemic has put them in the spotlight due to the negative impact linked to their alteration or loss and the high prevalence of long-term dysfunctions [35,36]. A meta-analysis by Tan et al. [14] highlighted the severity of long-term self-reported smell and taste alterations in individuals with COVID-19, with about 5% of patients developing persistent alterations, considering data from 3699 patients in 18 studies. Further, based on sensitivity analyses, the authors indicated that this rate could be an underestimate. The fact the COVID+ group in our sample, composed of participants affected by long-term complications of COVID-19, reported lower chemosensory abilities (smell, taste, and chemesthesis) compared to the COVID− group is in line with previous studies using both self-reported [6,13] and direct psychophysical [37,38] measures. The COVID+ group also reported reduced experienced intensities to smell and taste household items compared to the COVID− group, whereas no differences were found regarding chemesthetic stimuli. Furthermore, unlike previous studies using self-reports [3,6,13], we did not observe in our sample that the number of days since a COVID-19 diagnosis was a significant predictor for the outcome of chemosensory abilities and experienced intensities. Previous studies found that participants present a more profound impairment during the first two weeks of COVID-19 infection, with the most significant increase in the assessment of self-reported chemosensory abilities taking place between 10 and 20 days [18]. In this regard, it should be noted that, given the focus of the present study on long-term COVID-19, the participant enrollment was highly variable (days since COVID-19 diagnosis = 267.3 ± 113.9). This contrasts with previous studies, where participants were usually recruited shortly after the diagnosis of a COVID-19 infection [37].

Many studies have evaluated chemosensory dysfunction in people affected by COVID-19, primarily using self-reported measures, including surveys and remote interviews [36] due to the difficulty in contacting actively infected participants and conducting direct testing with them for fear of contagion. Although previous research has shown that direct and self-reported assessments do not always correspond to each other [19,20,21], including chemosensory measurements in participants affected by COVID-19 [4,22], here, we found a significant association between the two methods. The responses to the calibrated odor stimulus delivered via SCENTinel highly and positively correlated with the odor intensity collected via household items via the GCCR Smell and Taste Check as well as with self-reported smell ability on the day of testing. This opposes the view that self-reported (subjective) methods have a lower sensitivity than direct psychophysical (objective) tests of chemosensory alterations [39]. The discrepancies previously found between both methods could be related to the use of simple/reductionist questions (e.g., a binary response of “Yes/No” to “Can you smell?”) or unidimensional tests (e.g., a simple odor identification test). Such debate also relates to the intrinsic advantages and disadvantages of both approaches. Furthermore, the operational definition of recovery has also been shown to modulate the agreement between both methods. For instance, in the study of Prajapati et al. [23], self-reports underestimated recovery compared to direct assessments when defined as 10/10 on a smell function VAS. Interestingly, this discrepancy disappeared when recovery was defined as 8/10 on the same VAS.

A significant correspondence between self-reported and direct measures has also been reported in recent COVID-19 studies. For instance, Ciofalo et al. [37] used a VAS to assess olfactory–gustatory symptoms as a self-reported measure, the Brief Smell Identification Test [40], and used Burghart Taste Strips [41] as direct measures of smell and taste function. Self-reported smell loss (85.2%) corresponded positively with the smell identification outcome, supporting the idea that subjective and objective smell testing correlate well [42], at least in certain conditions. All in all, our findings align with studies showing that patients with self-reported impaired chemosensory abilities perform poorly on direct psychophysical measurements [43], calling for the inclusion, at a minimum, of rapid direct psychophysical testing. Although the assessment of chemosensory function by means of subjective measures has been questioned [44], our results speak in favor of the use of self-reports in individuals affected by long-term COVID-19, at least to identify their subjective experience of sensory loss or distortion. Furthermore, while direct methods to detect chemosensory dysfunction often involve a time-consuming and expensive endeavor, our findings suggest that the use of standardized odor stimuli that include the (unconventional) measurement of odor intensity—such as the SCENTinel test [27]—as well as unstandardized household items and self-reports of chemosensory abilities can help to overcome such limitations due to the possibility of being self-administered and their simple and low-cost administration.

Some of the strengths of this study relate to the inclusion of a relatively large sample size, considering both participants affected by COVID-19 and participants without COVID-19, as well as the joint acquisition of self-reported and direct measures of chemosensory function. Further, this is one of the few studies including three chemical senses, namely smell, taste, and chemesthesis, whose interaction has been suggested to be relevant for recovery [8]. Indeed, while smell and taste alterations are often reported as chemosensory alterations in the COVID-19 literature, few studies have included reports on chemesthetic ability. Furthermore, when it comes to the sense of smell, we aimed to go beyond odor identification by using a validated, low-cost test that could also screen for odor detection and intensity. This study is not free of limitations. Given the nonprobabilistic nature of the sampling used in this study, we cannot rule out a selection bias, given that it is possible that the individuals, both COVID+ and COVID−, that decided to participate in this study were particularly interested in chemosensory alterations due to the pandemic scenario. Indeed, all participants in the COVID+ group self-reported one or more chemosensory alteration. Although the presence of chemosensory dysfunction was not an inclusion criterion, this potential bias should be considered when generalizing our findings in light of the variable prevalence of chemosensory dysfunction in individuals affected by COVID-19 [17]. Another limitation of our study relates to the unbalanced samples for the COVID+ (*n* = 133) and COVID− (*n* = 75) groups due to the nonprobability convenience sampling. Future studies could benefit from including more balanced groups as well as matching sociodemographic criteria (e.g., sex and age). Furthermore, whereas this study accounted for three olfactory skills (i.e., detection, intensity, and identification), future studies could measure the participants’ olfactory thresholds. As pointed out by Trecca et al. [2], olfactory threshold performance is often reported to be more impacted than odor identification and discrimination as assessed via the Sniffin’ Sticks extended test [25]. Further studies should confirm these results in a group of participants formally diagnosed with chemosensory dysfunction (via clinical opinion or other gold-standard olfactometric tests).

Follow-up is necessary to assess the recovery trajectory of individuals with chemosensory alterations, particularly considering that participants tend to overestimate the persistence and recovery from chemosensory dysfunction when using self-reported methods [36]. Indeed, despite the vast literature on the diagnostic relevance of chemosensory dysfunction in COVID-19, inconsistent results regarding its clinical course call for further research. Furthermore, recent evidence suggests that chemosensory dysfunction is less likely with new COVID-19 variants [7]. Indeed, the Omicron variant has been observed to affect chemosensory function less severely [45,46]. Data from 294 participants who responded to a 6-month follow-up interview on the evolution of Omicron-related chemosensory dysfunction showed that 96% reported complete resolution at 6 months (95% CI 90.2–98.9%) [36]. As suggested by Tan et al. [14], the variability regarding the recovery rates might be associated with the underlying mechanisms of smell and taste alterations following COVID-19 infection. A recent study [47] proposed a molecular explanation for SARS-CoV-2-induced smell loss, suggesting a non-cell-autonomous mechanism of nuclear reorganization that downregulates olfactory receptors and their signaling genes in olfactory sensory cells.

All in all, our results showed that self-reports matched direct psychophysical assessments of chemosensory abilities, as assessed via SCENTinel and via the GCCR Smell and Taste Check, contributing to the discussion on the best practices to monitor chemosensory dysfunction in long-term COVID-19. This study supports the idea that direct and self-reported measures correspond well, highlighting the suitability of using both types of measures for chemosensory screening on large populations. Further, our findings corroborate that chemosensory dysfunction, comprising smell, taste, and chemesthetic alterations, is present in individuals affected by COVID-19. Importantly, direct measures (e.g., SCENTinel), even when self-administered at home, are crucial to understanding the different profiles of chemosensory impairment in long-term COVID-19 and can help to monitor the symptoms and recovery of participants.

## Figures and Tables

**Table 1:** Sociodemographic characterization of the sample by COVID-19 status.

Variable	COVID+ (*n* = 133)	COVID− (*n* = 75)	ANOVA/Chi-Squared Test
Age (years), mean ± SD	42.4 ± 13.9	38.6 ± 12.2	*F*(1, 173) = 3.51, *p* = 0.063, *η*^2^_p_ = 0.020
Days since COVID-19 diagnosis, mean ± SD	267.3 ± 113.9		
Gender, *n* (%)			χ^2^(1) = 7.9, *p* = 0.005, Cramér’s V = 0.195
Female	107 (80.4)	47 (62.7)	
Male	26 (19.6)	28 (37.3)	
Education (years), *n* (%)			χ^2^(3) = 63, *p* < 0.001, Cramér’s V = 0.552
8–14	32 (24.1)	2 (2.7)	
15–19	56 (42.1)	29 (38.7)	
≥20	14 (10.5)	42 (56.0)	
Not shared	31 (23.3)	2 (2.7)	
Socioeconomic status, *n* (%)			χ^2^(3) = 26, *p* < 0.001, Cramér’s V = 0.374
Upper	0 (0.0)	6 (11.8)	
Upper middle	45 (33.8)	19 (37.3)	
Lower middle	43 (32.3)	19 (37.3)	
Lower	1 (0.8)	2 (3.9)	
Not shared	44 (33.1)	5 (9.8)	
Smoking status, *n* (%)			χ^2^(3) = 16, *p* = 0.001, Cramér’s V = 0.273
Current smoker	13 (9.8)	11 (14.7)	
Former smoker	23 (18.8)	17 (22.7)	
Never smoker	64 (48.1)	45 (60.0)	
Not shared	31 (23.3)	2 (2,7)	
Vaccination status, *n* (%)			χ^2^(3) = 43, *p* < 0.001, Cramér’s V = 0.455
One dose	40 (30.1)	10 (13.3)	
Two doses	41 (30.8)	58 (77.3)	
Not vaccinated	11 (8.3)	3 (4.0)	
Not shared	41 (30.8)	4 (5.3)	

**Table 2:** Self-reported frequency of smell and taste alterations.

Type of Alteration, *n* (%)	COVID+
Smell	
Smell loss	105 (78.9)
Parosmia	86 (64.7)
Fluctuations	37 (27.8)
Phantosmia	36 (27.1)
Taste	
Taste loss	79 (59.4)
Dysgeusia	55 (41.3)
Fluctuations	21 (15.8)
Phantogeusia	9 (6.8)

Note: None of the COVID− participants reported chemosensory alterations. Chemesthesis was not assessed in the GCCR Smell and Taste Check as a binary response question. It was only assessed through VAS ratings of ability and intensity.

**Table 3:** Ratings of chemosensory abilities and the experienced intensity of household items via the GCCR Smell and Taste Check by COVID-19 status.

GCCR Smell and Taste Check Measure	COVID+	COVID−	ANOVA	Effect Size (*η*^2^_p_)
Self-reports of ability, mean ± SD				
Smell	42.6 ± 26.2	77.8 ± 18.7	*F*(1, 180) = 98, *p* < 0.001	0.353
Taste	56.9 ± 26.6	84.5 ± 18.0	*F*(1, 180) = 60.3, *p* < 0.001	0.251
Chemesthesis	61.2 ± 27.7	74.0 ± 20.5	*F*(1, 180) = 11.4, *p* < 0.001	0.060
Household item intensity, mean ± SD				
Smell	50.7 ± 26.6	65.6 ± 20.2	*F*(1, 180) = 16.5, *p* < 0.001	0.084
Taste	70.4 ± 20.0	76.1 ± 16.1	*F*(1, 180) = 4.03, *p* = 0.046	0.022
Chemesthesis	70.8 ± 24.5	74.8 ± 21.5	*F*(1, 177) = 1.27, *p* = 0.260	0.007

**Table 4:** Percentage of participants that passed SCENTinel by COVID-19 status.

SCENTinel Test, *n* (%)	COVID+	COVID−	Chi-Squared Test	Effect Size (Cramér’s V)
Detection	92 (69.2)	68 (90.7)	χ^2^(1) = 12, *p* < 0.001	0.245
Intensity	117 (88.0)	73 (97.3)	χ^2^(1) = 5, *p* = 0.020	0.160
Identification (1st attempt)	77 (57.9)	65 (86.7)	χ^2^(1) = 18, *p* < 0.001	0.297
Identification (2nd attempt)	18 (32.1)	9 (90)	χ^2^(1) = 12, *p* < 0.001	0.422
Total score	85 (63.9)	68 (90.7)	χ^2^(1) = 18, *p* < 0.001	0.291

**Table 5:** Null association between chemosensory dysfunction and time relative to COVID-19 onset.

Measure	*β*	*SE*	*z* Value	*p* Value	95% CI	
					Lower Limit	Upper Limit
GCCR self-reports of ability						
Smell	0.0156	0.0244	0.64	0.520	−0.0273	0.0586
Taste	0.0094	0.0235	0.40	0.690	−0.0347	0.0534
Chemesthesis	0.0125	0.0243	0.52	0.610		
GCCR household item intensity						
Smell	0.0260	0.0214	1.21	0.230	−0.0179	0.0699
Taste	0.0137	0.0201	0.68	0.500	−0.0194	0.0468
Chemesthesis	0.0189	0.0223	0.85	0.400	−0.0218	0.0596
SCENTinel test						
Detection	−0.0008	0.0005	−1.59	0.110	−0.0028	0.0010
Intensity	0.0004	0.0002	1.57	0.115	−0.0012	0.0019
Intensity rating ^1^	0.0036	0.0149	0.24	0.810	−0.0302	0.0373
Identification (1st attempt)	−0.0002	0.0007	−0.25	0.800	−0.0022	0.0018
Identification (2nd attempt)	−0.0009	0.0018	−0.51	0.612	−0.0053	0.0029
Total score	−0.0002	0.0006	−0.39	0.699	−0.0022	0.0016

^1^ The intensity rating refers to the numeric score reported by the participant and not to whether the participant met the accuracy criteria for the SCENTinel intensity subtest.

## Data Availability

The data and R scripts generated during the current study are available in the Open Science Framework repository accessible at https://osf.io/3xbqg/?view_only=5e3b7b32ab9147bc89bb72aefe4b79e5 accessed on 15 February 2022.

## References

[1] Giacomelli A., Pezzati L., Conti F., Bernacchia D., Siano M., Oreni L., Rusconi S., Gervasoni C., Ridolfo A.L., Rizzardini G. (2020). Self-reported olfactory and taste disorders in patients with severe acute respiratory coronavirus 2 infection: A cross-sectional study. Clin. Infect. Dis..

[2] Trecca E.M., Cassano M., Longo F., Petrone P., Miani C., Hummel T., Gelardi M. (2022). Results from psychophysical tests of smell and taste during the course of SARS-CoV-2 infection: A review. Acta Otorhinolaryngol. Ital..

[3] Parma V., Ohla K., Veldhuizen M.G., Niv M.Y., Kelly C.E., Bakke A.J., Cooper H.W., Bouysset C., Pirastu N., Dibattista M. (2020). More than smell—COVID-19 is associated with severe impairment of smell, taste, and chemesthesis. Chem. Senses.

[4] Mazzatenta A., Neri G., D’Ardes D., De Luca C., Marinari S., Porreca E., Cipollone F., Vecchiet J., Falcicchia C., Panichi V. (2020). Smell and taste in severe COVID-19: Self-reported vs. testing. Front. Med..

[5] Desai M., Oppenheimer J. (2021). The importance of considering olfactory dysfunction during the COVID-19 pandemic and in clinical practice. J. Allergy Clin. Immunol. Pract..

[6] Gerkin R.C., Ohla K., Veldhuizen M.G., Joseph P.V., Kelly C.E., Bakke A.J., Steele K.E., Farruggia M.C., Pellegrino R., Pepino M.Y. (2021). Recent smell loss is the best predictor of COVID-19 among individuals with recent respiratory symptoms. Chem. Senses.

[7] Coelho D.H., Reiter E.R., French E., Costanzo R.M. (2022). Decreasing Incidence of Chemosensory Changes by COVID-19 Variant. Otolaryngol. Head Neck Surg..

[8] Cecchetto C., Di Pizio A., Genovese F., Calcinoni O., Macchi A., Dunkel A., Ohla K., Spinelli S., Farruggia M.C., Joseph P.V. (2021). Assessing the extent and timing of chemosensory impairments during COVID-19 pandemic. Sci. Rep..

[9] Worldometer COVID-19 Coronavirus Pandemic. https://www.worldometers.info/coronavirus/.

[10] Herz R.S., Bajec M.R. (2022). Your money or your sense of smell? A comparative analysis of the sensory and psychological value of olfaction. Brain Sci..

[11] Stevenson R.J. (2010). An initial evaluation of the functions of human olfaction. Chem. Senses.

[12] Vaira L.A., Gessa C., Deiana G., Salzano G., Maglitto F., Lechien J.R., Saussez S., Piombino P., Biglio A., Biglioli F. (2022). The effects of persistent olfactory and gustatory dysfunctions on quality of life in long-COVID-19 patients. Life.

[13] Ohla K., Veldhuizen M.G., Green T., Hannum M.E., Bakke A.J., Moein S.T., Tognetti A., Postma E.M., Pellegrino R., Hwang D.L.D. (2022). A follow-up on quantitative and qualitative olfactory dysfunction and other symptoms in patients recovering from COVID-19 smell loss. Rhinology.

[14] Tan B.K.J., Han R., Zhao J.J., Tan N.K.W., Quah E.S.H., Tan C.J.W., Chan Y.H., Teo N.W.Y., Charn T.C., See A. (2022). Prognosis and persistence of smell and taste dysfunction in patients with COVID-19: Meta-analysis with parametric cure modelling of recovery curves. BMJ.

[15] Cao A.C., Nimmo Z.M., Mirza N., Cohen N.A., Brody R.M., Doty R.L. (2022). Objective screening for olfactory and gustatory dysfunction during the COVID-19 pandemic: A prospective study in healthcare workers using self-administered testing. World J. Otorhinolaryngol. Head Neck Surg..

[16] Hannum M.E., Ramirez V.A., Lipson S.J., Herriman R.D., Toskala A.K., Lin C., Joseph P.V., Reed D.R. (2020). Objective sensory testing methods reveal a higher prevalence of olfactory loss in COVID-19–positive patients compared to subjective methods: A systematic review and meta-analysis. Chem. Senses.

[17] Hannum M.E., Koch R.J., Ramirez V.A., Marks S.S., Toskala A.K., Herriman R.D., Lin C., Joseph P.V., Reed D.R. (2022). Taste loss as a distinct symptom of COVID-19: A systematic review and meta-analysis. Chem. Senses.

[18] Vaira L.A., Hopkins C., Salzano G., Petrocelli M., Melis A., Cucurullo M., Ferrari M., Gagliardini L., Pipolo C., Deiana G. (2020). Olfactory and gustatory function impairment in COVID-19 patients: Italian objective multicenter-study. Head Neck.

[19] Jang S.S., Choi J.S., Kim J.H., Kim N., Ference E.H. (2022). Discordance between subjective and objective measures of smell and taste in US adults. Otolaryngol. Head Neck Surg..

[20] Philpott C.M., Wolstenholme C.R., Goodenough P.C., Clark A., Murty G.E. (2006). Comparison of subjective perception with objective measurement of olfaction. Otolaryngol. Head Neck Surg..

[21] Rawal S., Hoffman H.J., Chapo A.K., Duffy V.B. (2014). Sensitivity and specificity of self-reported olfactory function in a home-based study of independent-living, healthy older women. Chemosens. Percept..

[22] Lechien J.R., Chiesa-Estomba C.M., Vaira L.A., De Riu G., Cammaroto G., Chekkoury-Idrissi Y., Circiu M., Distinguin L., Journe F., de Terwangne C. (2021). Epidemiological, otolaryngological, olfactory and gustatory outcomes according to the severity of COVID-19: A study of 2579 patients. Eur. Arch. Oto-Rhino-Laringol..

[23] Prajapati D.P., Shahrvini B., Said M., Srinivas S., DeConde A.S., Yan C.H. (2021). Assessment of patient recognition of coronavirus disease 2019 (COVID-19)-associated olfactory loss and recovery: A longitudinal study. Int. Forum Allergy Rhinol..

[24] Doty R.L., Shaman P., Dann M. (1984). Development of the University of Pennsylvania Smell Identification Test: A standardized microencapsulated test of olfactory function. Physiol. Behav..

[25] Hummel T., Sekinger B., Wolf S.R., Pauli E., Kobal G. (1997). “Sniffin” sticks.’ Olfactory performance assessed by the combined testing of odor identification, odor discrimination and olfactory threshold. Chem. Senses.

[26] Doty R.L. (2018). Measurement of chemosensory function. World J. Otorhinolaryngol. Head Neck Surg..

[27] Parma V., Hannum M.E., O’Leary M., Pellegrino R., Rawson N.E., Reed D.R., Dalton P.H. (2021). SCENTinel 1.0: Development of a rapid test to screen for smell loss. Chem. Senses.

[28] Duff K., McCaffrey R.J., Solomon G.S. (2002). The Pocket Smell Test: Successfully discriminating probable Alzheimer’s dementia from vascular dementia and major depression. J. Neuropsychiatry Clin. Neurosci..

[29] Rawal S., Hoffman H.J., Honda M., Huedo-Medina T.B., Duffy V.B. (2015). The taste and smell protocol in the 2011–2014 US National Health and Nutrition Examination Survey (NHANES): Test–retest reliability and validity testing. Chemosens. Percept..

[30] World Medical Association (2013). WMA Declaration of Helsinki: Ethical principles for medical research involving human subjects. JAMA.

[31] Arslan R.C., Walther M.P., Tata C.S. (2020). formr: A study framework allowing for automated feedback generation and complex longitudinal experience-sampling studies using R. Behav Res. Methods.

[32] Zou G. (2004). A modified poisson regression approach to prospective studies with binary data. Am. J. Epidemiol..

[33] Croy I., Buschhüter D., Seo H.S., Negoias S., Hummel T. (2010). Individual significance of olfaction: Development of a questionnaire. Eur. Arch. Oto-Rhino-Laryngol..

[34] Trecca E.M., Fortunato F., Gelardi M., Petrone P., Cassano M. (2021). Development of a questionnaire to investigate socio-cultural differences in the perception of smell, taste and flavour. Acta Otorhinolaryngol. Ital..

[35] Boscolo-Rizzo P., Polesel J., Vaira L.A. (2022). Smell and taste dysfunction after COVID-19. BMJ.

[36] Boscolo-Rizzo P., Tirelli G., Meloni P., Hopkins C., Lechien J.R., Madeddu G., Cancellieri E., Lazzarin C., Borsetto D., De Vito A. (2022). Recovery from COVID-19 related olfactory and gustatory dysfunction following omicron BA. 1 subvariant infection: A six-month prospective study. Res. Sq..

[37] Ciofalo A., Cavaliere C., Masieri S., Di Chicco A., Fatuzzo I., Lo Re F., Baroncelli S., Begvarfaj E., Adduci A., Mezzaroma I. (2022). Long-Term Subjective and Objective Assessment of Smell and Taste in COVID-19. Cells.

[38] Lechien J.R., Chiesa-Estomba C.M., Beckers E., Mustin V., Ducarme M., Journe F., Marchant A., Jouffe L., Barillari M.R., Camarotto G. (2021). Prevalence and 6-month recovery of olfactory dysfunction: A multicentre study of 1363 COVID-19 patients. J. Intern. Med..

[39] von Bartheld C.S., Hagen M.M., Butowt R. (2020). Prevalence of chemosensory dysfunction in COVID-19 patients: A systematic review and meta-analysis reveals significant ethnic differences. ACS Chem. Neurosci..

[40] Doty R.L., Marcus A., Lee W.W. (1996). Development of the 12-item Cross-Cultural Smell Identification Test (CC-SIT). Laryngoscope.

[41] Landis B.N., Welge-Luessen A., Bramerson A., Bende M., Mueller C.A., Nordin S., Hummel T. (2009). “Taste Strips”—A rapid, lateralized, gustatory bedside identification test based on impregnated filter papers. J. Neurol..

[42] Seok J., Shim Y.J., Rhee C.S., Kim J.W. (2017). Correlation between olfactory severity ratings based on olfactory function test scores and self-reported severity rating of olfactory loss. Acta Otolaryngol..

[43] Bertlich M., Stihl C., Lüsebrink E., Hellmuth J.C., Scherer C., Freytag S., Spiegel J.L., Stoycheva I., Canis M., Weiss B.G. (2021). The course of subjective and objective chemosensory dysfunction in hospitalized patients with COVID-19: A 6-month follow-up. Eur. Arch. Otorhinolaryngol..

[44] Lötsch J., Hummel T. (2019). Clinical usefulness of self-rated olfactory performance—A data science-based assessment of 6000 patients. Chem. Senses.

[45] Boscolo-Rizzo P., Tirelli G., Meloni P., Hopkins C., Madeddu G., De Vito A., Gardenal N., Valentinotti R., Tofanelli M., Borsetto D. (2022). Coronavirus disease 2019 (COVID-19)–related smell and taste impairment with widespread diffusion of severe acute respiratory syndrome–coronavirus-2 (SARS-CoV-2) Omicron variant. Int. Forum Allergy Rhinol..

[46] Dehgani-Mobaraki P., Patel Z., Zaidi A.K., Giannandrea D., Hopkins C. (2022). The Omicron Variant of SARS-CoV-2 and its Effect on the Olfactory System. Int. Forum Allergy Rhinol..

[47] Zazhytska M., Kodra A., Hoagland D.A., Frere J., Fullard J.F., Shayya H., McArthur N.G., Moeller R., Uhl S., Omer D.A. (2022). Non-cell-autonomous disruption of nuclear architecture as a potential cause of COVID-19-induced anosmia. Cell.

